# Novel circulating- and imaging-based biomarkers to enhance the mechanistic understanding of human drug-induced liver injury

**Published:** 2017-02-12

**Authors:** Joanna I Clarke, Nathalie Brillanf, Daniel J Antoine

**Affiliations:** Department of Molecular & Clinical Pharmacology, MRC Centre for Drug Safety Science, Institute of Translational Medicine, University of Liverpool, Liverpool, United Kingdom

**Keywords:** miR-122, HMGB1, K18, qualification, apoptosis, necrosis, inflammation, function, MSOT, photoacoustic, MRI

## Abstract

Liver safety biomarkers in current clinical practice are recognized to have certain shortcomings including their representation of general cell death and thus lacking in indicating the specific underlying mechanisms of injury. An informative panel of circulating- and imaging-based biomarkers, will allow a more complete understanding of the processes involved in the complex and multi-cellular disease of drug-induced liver injury; potentially preceding and therefore enabling prediction of disease progression as well as directing appropriate, existing or novel, therapeutic strategies. Several putative liver safety biomarkers are under investigation as discussed throughout this review, informing on a multitude of hepatocellular mechanisms including: early cell death (miR-122), necrosis (HMGB1, K18), apoptosis, (K18), inflammation (HMGB1), mitochondrial damage (GLDH, mtDNA), liver dysfunction (MRI, MSOT) and regeneration (CSF1). These biomarkers also hold translational value to provide important read across between in vitro-in vivo and clinical test systems. However, gaps in our knowledge remain requiring further focussed research and the ultimate qualification of key exploratory biomarkers.

**Relevance for patients:** this novel multi-modal approach of assessing drug-induced liver injury could potentially enable better patient stratification and enhance treatment strategies. Ultimately, this could reduce unnecessary treatment, also decreasing hospital bed occupancy, whilst ensuring early and accurate identification of patients needing intervention.

## 1. Introduction

Associated with more than 10 % of documented human medicines, drug-induced liver injury (DILI) is of widespread concern throughout sectors - impacting drug safety scientists, clinicians and patients. Human DILI is a complex event comprised of many underlying processes [1,2]. It is therefore often difficult to link causal pathophysiology with clinically measurable characteristics. Accurate diagnosis of DILI is important but known to be challenging and hepatic injury of this kind is still generally considered a diagnosis of exclusion [3].

Acetaminophen (APAP or paracetamol) poisoning is a major contributor to this, particularly in industrialized countries where it is the most common acute overdose seen, causing over 500 deaths per year in the United States due to both accidental and intentional aetiologies [4,5]. The pharmacology of APAP is well documented and it is commonly used as a hepatotoxicity model, to study liver injury. It is the highly reactive metabolite NAPQI (N-acetyl-*p*-benzoquinone imine) which is responsible for cellular injury in supra-therapeutic exposure [6]. At therapeutic doses (4g/day) [7], APAP undergoes detoxification in the liver by phase II enzymes but in overdose situations, the glucuronidation and sulphation pathways are overwhelmed, and a larger portion of APAP is metabolized through phase I (CYP2E1) [8-10] to NAPQI [6,11]. NAPQI is conjugated with glutathione (GSH) [12]; however GSH levels are limited and once depleted below a critical level NAPQI is free to react with cellular macromolecules [13] and covalently bind to cellular proteins, increase ROS (reactive oxygen species) [6] and disrupt mitochondrial function (being one of the key outcomes) [14], which consequently leads to hepatocellular injury and necrotic changes mainly observed in the centrilobular regions [15].

Serum biomarkers alanine aminotransferase (ALT) and total bilirubin (TBL) have been in clinical use for many years and still remain a part of the gold standard in identifying DILI. As a result, their behaviour has been comprehensively studied and numerous shortcomings have been identified [16]. Serum ALT and TBL elevation are currently established as Hy’s Law to identify serious liver injury according to the US Food and Drug Administration (FDA) [17]. However, the methods used to quantify ALT activity have not been standardized and a robust definition of normal reference ranges has not been agreed upon; these ranges inevitably depend upon the population group defined as normal and assay measurements will vary between laboratories. Although ALT activity is regarded as generally sensitive for detecting liver injury when it occurs, it is not sensitive with respect to time/kinetics. Furthermore, ALT activity has often been described as having little prognostic value due to the fact that an ALT elevation represents probable injury to the liver after it has occurred. From a regulatory point of view, elevations in ALT activity are also worrisome with regards to establishing liver safety during drug treatment. Frequent and relatively large elevations in ALT activity are associated with treatments that do not pose a clinical liver safety issue, such as low dose APAP, heparins and tacrine [18,19]. The challenge here is to distinguish between benign elevations in ALT activity and the potential for a serious DILI outcome.

Regarding circulating TBL, this biomarker for liver function only appears in blood when there is an advanced liver damage frequently leading to late diagnosis and subsequently undesired management and treatments. Largely, these include a lack of tissue specificity and sensitivity (delayed elevations) resulting in an unacceptable frequency of false positive/negative results and absence of informative mechanistic information [20-22]. The development of informative biomarkers of DILI therefore remains a primary aim in clinical and pre-clinical settings [20]. Despite these shortcomings, the combined approach of ALT and TBL represent the current standard any novel biomarker must surpass to provide added value.

**Figure 1.**
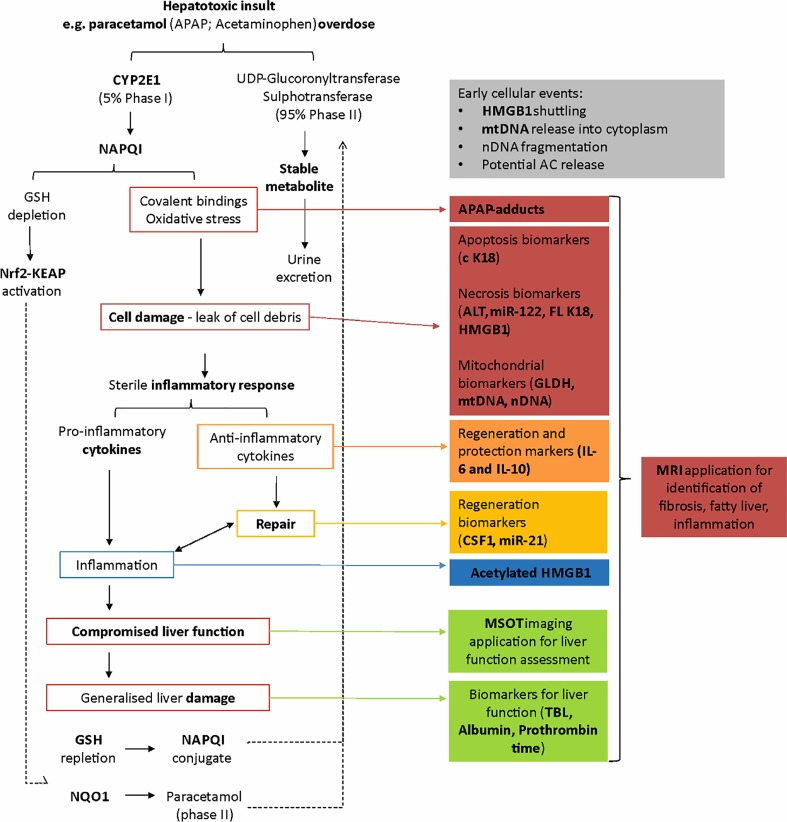
Flow diagram represents the presence and utility of exploratory drug-induced liver injury biomarkers using APAP-induced overdose as a model hepatotoxic process and focussing on the mechanistic insight gained from the interpretation of each marker.

The definition of a biomarker has now been widely cited and their worth widely recognised as minimally invasive means of working out the processes underlying injuries such as DILI. An improved biomarker must add utility or value to currently used diagnostics in order to achieve validity and widespread acceptance [23]. Novel liver biomarkers must therefore be shown to improve patient safety (in the general sense of adding to risk-benefit assessment) or add mechanistic value (aid therapy stratification or the design of new drug targets) [24]. It is thought that combinations or ‘panels’ of circulating biomarkers may be likely to surpass the utility of one marker alone as they are able to complement advantageous features and create the ultimate working profile (Figure 1) [24].

Markers that give an enhanced mechanistic insight are likely to play a key role in this putative panel. Primarily, by informing on the pathophysiology of injury and identifying an exact mechanism, biomarkers of this kind could by their nature be extremely useful in directing appropriate therapeutic interventions to specific targets and hereby allowing treatment in a stratified and personalised manner [25,26]. In addition, it is logical that mechanistic biomarkers will coincide with the earlier detection of liver injury as the mechanisms induced will inherently preside injury itself and any successive clinical symptoms [25,27]. Finally, biomarkers informing of a mechanism of injury could theoretically hold a characteristic and direct quantitative association with extent of injury, again allowing for improved patient stratification or a more informed evaluation of risk-benefit [27].

Biomarkers can be utilised in numerous settings within basic research, drug development and clinical practice. It is for this reason that the translatability of biomarkers is a desired attribute but often overlooked. Knowledge of biomarker conservation between models/species is incredibly important and a biomarker that is translational between *in vitro* models, animals and humans would certainly aid application within drug development. However, it has been suggested that a marker being translational between only rodent models and humans would be acceptable [28]. Throughout this review we will place focus on the emerging need for a biomarker, or indeed a panel of biomarkers, which allow a mechanistic insight into injury to be gained (referred to as a ‘mechanistic biomarker’).

## 2. Hepatic injury markers

### 2.1. microRNA-122 (miR-122)

microRNAs (miRNAs), short (18-25 nucleotides) and noncoding RNA molecules, have become of great interest in numerous research fields and a variety of pathological settings [20]. They function to repress or negatively regulate specific cellular proteins and thus impact the phenotype of the cell [20]. Additionally, circulating miRNAs are accessible (via the blood), stable, many show organ specificity and translational conservation thus providing advantageous biomarkers for a multitude of therapeutic settings [29]. In hepatological terms, miRNAs are of significance, playing a critical role in normal liver development and fine-tuning fundamental biological liver processes [30].

miR-122 is perhaps the most widely-investigated of the putative DILI miRNA markers; thought to be important in cholesterol metabolism, hepatitis C virus (HCV) infection and hepatocellular carcinoma (HCC). However, its role within each of these processes varies considerably; regarded as holding a major positive role in HCV, being likely to facilitate in virus replication, and by contrast holding a negative role in HCC, displaying downregulated levels in these scenarios [31]. miR-122 is widely conserved across species, has been shown to be more sensitive than traditional marker ALT, and exhibits exclusive hepatic expression (constituting over 70% of total hepatic miRNA) rendering miR-122 as a translational, sensitive and organ specific biomarker, respectively [29]. The translational value of miR-122 has not only been shown between humans and rodents, but also in zebrafish where expression was found to be localized to the cytoplasm of hepatocytes and subsequently measurable in blood using the same assay as for clinical serum samples [32]. Possibly most striking of the studies placing focus on miR-122, is that of a recent published case report identifying that miR-122 could have detected oncoming liver injury at an earlier time point and thus potentially avoided life-threatening hepatotoxicity following an APAP overdose [33].

Despite increasing confidence in the sensitivity and utility of miR-122 as a DILI biomarker, the precise mechanism of early release is not fully understood. Generally, the release of miRNAs into extracellular space and subsequently into the circulation is thought to occur either via binding to proteins (such as argonaute2 or lipoproteins) or bound within extracellular vesicles (exosomes, microparticles or larger apoptotic bodies). It may be that these varying mechanisms of release, prior to detection in the serum, are representative of differential underlying processes but comprehension of this remains incomplete [34]. It is known that hepatocyte-derived exosomes (HDEs) are able to readily cross fenestrations in the sinusoidal endothelium and enter the bloodstream. Clinically relevant alterations in the content of these exosomes have been reported in numerous models of liver injury where exosomes have been found to be immunomodulatory and HDE-based miR-122 has been shown to activate recipient monocytes [35]. Ultimately, the content of miR-122 in both exosomal and protein-rich compartments is increased following APAP administration. Distribution of miR-122 between these sections has been shown, by Holman et al., to alter temporally with exosomal miR-122 being found to decrease over time in high dose APAP studies. The authors here proposed biological signalling purposes, such as monocyte priming, as the cause of such changes in miR-122 plasma distribution [35].

### 2.2. Beyond miR-122?

Wider miRNA profiles have also been carried out not only identifying miR-122 and miR-192-5p (also formerly discussed in relevant literature), but additionally reported multiple members of the microRNA-200 and -101 families, miR-802-5p and miR-30d-5p as being consistently elevated during hepatobiliary injury in rats [36]. Moreover, in APAP overdose patients, other miRNA profiles have been identified as serum biomarkers able to discriminate between injury and non-injury. Alongside miR-122, miR-27b-3p and miR-21-5p, were also found to be elevated and report liver injury preceding ALT elevations. miR-192-5p was found to be inconsistent in this case but a panel of 11 miRNAs was found to be a valuable and informative tool able to provide early diagnosis and discriminate APAP hepatotoxicity from ischemic hepatitis, predict outcome and advocate accurate treatment [4]. In addition, also in human serum, 75 miRNA species have been found 3-fold or more increased and 46 species to be 3-fold or more decreased following APAP toxicity (from a total pool of 1,809 miRNA species) [37]. Indeed, miR-122 was among those found to display the largest increase along with miR-885-5p and miR-151a-3p. miR-122 was found to exhibit highest specificity, particularly when combined with miR-483-3p (a species found to decrease with injury) [37].

## 3. Cell death markers: necrosis

### 3.1. High mobility group box protein 1 (HMGB1)

Identified over 30 years ago, HMGB1, a ubiquitously expressed and nuclear protein has since been investigated within numerous pathologies [38]. HMGB1 is highly conserved between mammalian species and its structure and behaviour between the intracellular (both nuclear and cytosolic) and extracellular compartment has been defined very well elsewhere [39]. The localization and function of HMGB1 are known to be determined by post-translational modifications [40]. Within the nucleus, HMGB1 is involved in gene transcription interacting with nucleosome complexes [33,41-44]

Cells undergoing necrosis passively release a non-acetylated HMGB1 (fully reduced HMGB1) isoform which acts as a neutrophil chemoattractant. HMGB1 can then act as a damage associated molecular pattern (DAMP) by targeting Toll-like receptors (TLRs) and the receptor for advanced glycation end products (RAGE) within monocytes and macrophages; playing an important role in inducing an inflammatory response [45,46]. Once the inflammatory cells are activated they can liberate ROS. In oxidizing conditions, fully reduced HMGB1 can be oxidized to disulphide HMGB1 which will act as a turnover, as disulphide HMGB1 has higher affinity for TLR and RAGE, increasing cytokine release and inducing more inflammatory cell recruitment [39]. The disulfide and reduced cysteine form of HMGB1 are mutually exclusive, therefore the redox status of HMGB1 determines the shift between chemoattractant and inflammation, thus driving the function of HMGB1. It is yet unknown whether this effect is ubiquitous or limited to just APAP-induced injury and Gal/LPS [47].

Clinically, the prognostic utility has already been demonstrated and patients with APAP-induced hepatotoxicity show serum levels of total HMGB1 correlating strongly with ALT activity and prothrombin time, and holding association with a poor prognosis and outcome [48]. Pre-clinically, hepatocyte specific HMGB1 ablation has been shown to result in 100 % survival following an APAP dose that is usually lethal in mice [47]. Furthermore, studies have shown that anti-HMGB1 antibodies and knocking out *Hmgb1* in the liver reduces hepatic inflammation and liver injury in mouse models of APAP poisoning [43,45,49]. Anti-HMGB1 antibodies are in development for the treatment of human disease [28]. However, Jaeschke et al. reviewed in 2012 the role of the inflammatory response in APAP toxicity highlighting the beneficial impact by limiting the formation of pro-inflammatory mediators and by promoting repair of tissue damage [50].

As well as conventional DILI, HMGB1 has been shown to display increased expression and translocation correlating with disease stage in patients with alcoholic liver disease (ALD) [51]. HMGB1 has been shown to participate in the pathogenesis of ALD as the hepatocyte-selective ablation of *Hmgb1* in fact protected mice from alcohol-induced liver injury [51].

### 3.2. Keratin-18 (K18)

K18 is a type I intermediate filament protein expressed in epithelial cells and responsible for cell structure and integrity [52]. K18 constitutes approximately 5% of total hepatic protein [29] and two forms of this protein have been identified as novel and more sensitive biomarkers of DILI detectable in circulation. The first, full length (FL K18) version is passively released during necrotic cell death and hereby a marker of necrosis found to be elevated in multiple liver diseases such as non-alcoholic steatohepatitis (NASH) and hepatitis C infection as well as APAP toxicity [29]. Pilot studies have shown the ability of K18 to predict the development of ALI at first presentation to hospital following APAP overdose, with high sensitivity and specificity (ROC-AUC of 0.94 in comparison to 0.54 for ALT) [53]. Furthermore, clinical studies have also reported K18 as a biomarker for the therapeutic drug monitoring of chemotherapeutic agents [54].

Detection of circulating HMGB1 and K18 have also been used to infer the mechanisms of cell death in liver injury that is not drug-induced [55]. Elevated levels of circulating HMGB1, acetylated HMGB1 and K18 (keratin-18) were identified in the serum of patients with cholestatic liver injury (n=17), compared to patients without any concurrent injury (n=10), confirming inflammatory necrosis as an underlying mechanism of obstructive cholestasis [55]. This was previously demonstrated in a pre-clinical rodent study where plasma levels of K18, miR-122 and HMGB1 increased progressively after bile duct ligation (cholestasis) while caspase-cleaved cytokeratin-18 fragments (c K18) did not increase, indicating cell necrosis and the absence of apoptosis [56].

## 4. Biomarkers of inflammation

### 4.1. Acetylated HMGB1

The vital role of HMGB1 was demonstrated when Calogero et al. developed a *Hmgb1* -knock out mouse model by conventional gene targeting [57]. Their experiment indicated that *in vivo*, HMGB1 was essential for the survival of mice soon after birth, but *in vitro*, was not essential for the survival of cells or their proliferation [57]. HMGB1 has been previously identified as a precursor and mediator of the inflammatory response [43]. Inflammatory cells (monocytes and macrophages), can undergo HMGB1-acetylation after activation by inflammatory stimuli, leading to protein translocation to the cytoplasm and subsequent excretion to the extracellular compartment [58]. The two different isoforms of HMGB1 mentioned throughout this review are displayed in different temporal profiles allowing differentiation and classification of cell injury and inflammatory event as acetylated HMGB1 is not released from nonimmune cells [39].

Other mechanistic biomarkers have been highlighted from this inflammatory cascade. For instance, inflammatory mediators such as tumour necrosis factor and interferon have been implicated in increased susceptibility to APAP hepatotoxicity, whereas interleukins (IL)-6 and IL-10 have been implicated in hepatic regeneration and protection after a toxic insult [43].

To further investigate the role of HMGB1 in the coordination of the inflammatory response, there has recently been a focus in pre-clinical models on therapeutic interventions to inhibit HMGB1 release and function by using different strategies including exogenous agents (anti-HMGB1 antibody), small molecules inhibitors of HMGB1 and endogenous pep-tides [39,43]. Neutralizing circulating HMGB1 has been demonstrated to prevent inflammatory cell recruitment and to increase survival after APAP challenge [43].

It is very well known that the inflammatory response plays an essential role in the development and severity of liver injury induced by a drug. However, most pre-clinical and clinical models mentioned throughout this review involve acute toxicity with high doses of a drug, therefore omitting idiosyncratic DILI (IDILI) which accounts for up to 13 % of all acute liver failure (ALF) and represents a burden for drug development as it is ever difficult to predict IDILI [17,59]. IDILI has been widely described as an immune-mediated adverse drug reaction associated with human leukocyte antigen (HLA) and the principal reason that it is poorly understood is because pre-clinical animal models are lacking [60,61]. The mouse model currently used is known to develop immune tolerance and therefore subjects do not develop serious liver injury, as is the case for some patients that develop ALF in IDILI situations. Metushi et al. suggested that developing a model that has its immune tolerance inhibited could allow us to better understand this phenomena in human [61]. Nowadays, the prevention of IDILI is based on high-throughput genotyping and collaborative databases to evaluate the predisposition of each individual patient [62].

## 5. Cell death markers: apoptosis

### 5.1. Caspase-cleaved keratin-18 (c K18)

During apoptosis, the aforementioned FL K18 can be cleaved in a caspase-mediated process leaving caspase-cleaved K18 (c K18) to be the second form detectable in the serum [63]. Caspase-mediated cleavage of K18 is an early event in cellular structural rearrangement during apoptosis [64]. Caspases 3, 7 and 9 have been implicated in the cleavage of K18 at the C-terminal DALD/S motif.

Circulating FL K18 and c K18 have been shown to represent indicators of hepatic necrotic and apoptotic events, respectively, in mouse models of APAP induced liver injury [63] and during heparin-induced hepatocellular injury in man [19]. The prognostic utility of K18 has also been demonstrated in clinical DILI and acute liver injury [48]. In patients (n=78) with established acute liver injury following APAP overdose, elevations in absolute levels of necrosis K18 associate with a poor prognosis (indicated via the King’s College Criteria) and outcome and a total percentage of K18 attributed to apoptosis associates with improved survival [48].

### 5.2. HMGB1

HMGB1 has also been previously related to the apoptotic pathway. During early apoptosis, HMGB1 is not released and is sequestered in the nucleus but during late apoptosis/secondary necrosis, HMGB1 release is observed; however, this HMGB1 form lacks proinflammatory activity [45,65]. In immortalized cell lines, disulphide HMGB1 can translocate and regulate autophagy through Beclin1 binding or through signalling at RAGE, and nuclear HMGB1 modulates heat shock protein b-1 expression responsible for the coordination of mitophagy [66,67]. Therefore, the redox state of HMGB1 reglates programmed mechanisms of cell survival (autophagy) or cell death (apoptosis) in cancer cells [68].

## 6. Markers of mitochondrial damage

Recent efforts have also placed focus in developing a biomarker that is able to identify mitochondrial toxicity. DILI has previously and often been associated with mitochondrial dysfunction. Particularly, previous research into APAP toxicity revealed that NAPQI frequently binds mitochondrial proteins leading to mitochondrial oxidative stress and in turn to changes in the morphology and function of liver mitochondria that are detrimental to the cell [69]. Overall, studies in this area have thus far identified the mitochondrial matrix enzyme glutamate dehydrogenase (GLDH), mitochondrial DNA (mtDNA), nuclear DNA (nDNA) fragments and circulating acylcarnitines (ACs) [70,71].

### 6.1. Glutamate dehydrogenase (GLDH)

GLDH is elevated in both pre-clinical models and clinical cases of DILI and liver impairment highlighting its potential as a translational biomarker [53,69]. GLDH is an enzyme present in matrix-rich mitochondria (liver) and not in cristae-rich mitochondria (cardiac and skeletal muscle). GLDH is considered relatively liver-specific; additionally expressed in the brain and kidneys, but its release from these tissues is thought to enter the cerebrospinal fluid and tubular lumen respectively, rather than the blood [72,73]. Serum GLDH has been shown to be an early indicator of later ALT levels and hereby a more sensitive indicator of DILI [53,74].

Mechanistically, GLDH is thought to provide an indicator of leakage of mitochondrial contents into the circulation [75]. However, due to its relative large size (330 kDa), release of GLDH into the circulation is delayed during hepatocellular necrosis and therefore additionally provides an indicator of necrotic cell death. There yet remains some uncertainty as to whether measurement of GLDH could be useful in distinguishing benign elevations in ALT from those that portent severe DILI potential [21].

Studies from 2013 also found mitochondrial enzyme argininocuccinate synthetase to be increased in acute liver injury mouse models [70]. Upon comparison to ALT and AST, this enzyme along with sulfonerase isoform SULT2A1 were found to be superior in a variety of liver pathologies [70].

### 6.2. Mitochondrial DNA mtDNA (and nDNA fragments)

We know from rodent models that reactive species such as NAPQI are able to cause mitochondrial membrane permeability transition, pore opening, matrix swelling and outer membrane lysis in rodent models. This in turn is responsible for release of apoptosis-inducing factor and endonuclease G from mitochondria which translocate to the nucleus and consequently cause nuclear DNA fragmentation [69]. By contrast, our understanding of these mechanisms in humans is limited [69]. There is evidence for a stress on GSH levels after APAP exposure and APAP protein adducts are measurable in serum after overdose indicating that metabolic activation and protein adduct formation do occur in humans in a manner similar to that described in rodent models [26,69].

Recent research has aimed to gain further insight into the mechanisms of human APAP-induced liver injury with a wider goal of improving treatment prospects. In 2012, McGill et al. sought to assess whether mitochondrial injury and nDNA fragmentation play a part in the mechanism via the assessment of mtDNA levels and nDNA fragments measurable in the serum [69]. They were able to conclude that mitochondrial damage is central to APAP-induced cell death in murine models and in a human hepatocyte cell line and additionally that it is likely that mitochondrial dysfunction is a main determinant of liver cell damage in APAP overdose patients [69]. It has been previously reported that nuclear DNA fragments and mtDNA can act as DAMPs through activation of TLRs and induction of cytokine formation. Therefore, these molecules are involved in contributing to the activation of innate immune cells and removal of necrotic cell debris and recovery (as observed in mice). If the same mechanisms are true of human DILI, it can be deduced that mitochondrial dysfunction and DNA damage are critical events in mechanism of cell necrosis after APAP overdose in patients [69]. Importantly, this publication utilized furosemide (a hepatotoxin that does not affect mitochondrial function) in mice, showing significant elevations in ALT in these cases but only marginal increases in GLDH and mtDNA and therefore concluding that high levels of mtDNA and GLDH are specifically associated with this mechanism of toxicity and are not simply a result of tissue necrosis [69]. Thus, supporting the belief that these contents are released into the cytosol at an earlier stage of injury but only detectable in the plasma after the resulting hepatocellular necrosis [69].

In 2014, McGill et al. indeed went on to conclude that APAP overdose patients with more mitochondrial damage are less likely to survive, proving mitochondria to be central in the mechanism of APAP hepatotoxicity. GLDH, mtDNA and nDNA fragments were in fact all shown to be significantly increased in serum from ALF patients that died in comparison to those that survived. Importantly, ALT values were found to be similar between the two groups [26]. Circulating mtDNA levels have also been researched in other fields and found to be increased in response to stimuli such as trauma, improving risk prediction beyond commonly used biomarkers and having involvement in the development of inflammatory response syndromes [76,77].

Collectively, these data highlight the translational aspect of these mitochondrial biomarkers, reflecting their ease of utility in pre-clinical drug development studies as well as in clinical assessments - a key advantage. However, disadvantages exist-due to overlapping in the parameter values between survivors and non-survivors and subsequently low ROC-AUC values (a measure of sensitivity at 90% specificity) of these markers they remain to be individually considered as of limited clinical utility.

### 6.3. Acylcarnitines (ACs)

GLDH and mtDNA could be criticized for their relative size; as they are both too large to be detectable in serum until cell death or indeed loss of membrane integrity. ACshowever are more likely to represent markers that are detectable at even earlier time points, due to their early accumulation post-mit-ochondrial impairment [27,71]. ACs are derivatives of long-chain fatty acids required for the transport of these fatty acids into mitochondria for subsequent p-oxidation [71]. It has been demonstrated, that circulating ACs could be specific biomarkers of mitochondrial dysfunction in rodent models and potentially patients (although it is of note that patient serum levels did not appear raised in this instance) and hereby present as mechanistic DILI biomarkers [71]. The absent rise of ACs in APAP overdose patients is most likely due to the standard-of-care treatment with antidote N-acetylcysteine (NAC). It would be useful to study the behaviour of ACs in patient serum before NAC treatment commences or from patients suffering from alternative forms of DILI because an early, mechanistic biomarker of this kind would be an incredible attribute to clinical practice, potentially allowing a treatment decision to be made before injury progresses too far. Interestingly, a previous study demonstrated a significant rise in ACs in hospitalized children with APAP toxicity receiving delayed treatment with NAC, thus providing a functional and circulating biomarker associated with mitochondrial dysfunction [78]. Future studies should be applied to further investigate the clinical significance of AC elevations following APAP exposure and the role of ACs in any other condition known to be associated with impaired mitochondrial function.

## 7. Quantitative assessment of liver function

To date, in clinical scenarios, the panel of biomarkers utilized to diagnose DILI is based on Hy’s law alongside serum albumin and prothrombin time (markers of liver function) to provide a fuller picture of the status of the liver [39,78]. Even though the biomarkers mentioned above have been recognized as fundamental to efforts in translational hepatotoxicity research, many of the reported candidate biomarkers for hepatic drug safety have focused on hepatocyte injury rather than liver function, are still indicators of late stage liver injury, are not always specific to impaired liver and can be affected by other factors - therefore not suitable for early diagnosis and appropriate for treatment implementation.

Additionally in pre-clinical drug development, final assessment of DILI relies upon histological examination in combination with a panel of serum biomarkers [79]. However these biomarkers present different weaknesses and liver biopsy is still considered as the gold standard for the assessment of liver disease such as non-alcoholic fatty liver disease (NAFLD) or DILI. Due to its invasiveness, efforts have been dedicated in developing more suitable tools for the early and non-invasive diagnosis of liver injury in both pre-clinical and clinical environments. Imaging technologies have become of great interest to maintain experiments covering the NC3Rs and also to allow screening of large numbers of subjects at risk, or for follow-up of patients after therapeutic intervention.

### 7.1. Magnetic resonance imaging (MRI)

MRI is one of the methods that have been used to quantify liver injury [80]. To date it is the most accurate and reliable method of quantifying liver fat, liver fibrosis and liver inflammation with greater sensitivity and specificity compared to other technologies [80]. MRI can be used for longitudinal follow-up of patients and allows differentiation of the varying stages of diseases and lesions within the liver [81]. It is used with contrast reagents, such as gadolinium, and is applicable to measure liver fibrosis [80]. Extracellular uptake of gadolinium-based contrast agents (GBCA) in hepatic fibrosis has been shown to correlate with the early histological stage of fibrosis [82].

More recently, methods have been developed to measure liver stiffness allowing the assessment of inflammation and fibrosis in patients. MR elastography has been shown to derive stiffness measures that correlate with the different histological stages of fibrosis and this system is now commercially available and widely used clinically [83]. Another technique has used a combination of contrast reagents such as GBCA and hepatocyte uptake agent gadoxetate disodium to differentiate between regenerative liver and fibrotic liver [84].

Each imaging method has its own advantages and disadvantages; The majority of these techniques are applicable for the diagnosis of liver injury and some of them applicable for prognosis but not for the real assessment of liver function.

### 7.2. Multispectral optoacoustic tomography (MSOT)

The liver is one of the most complex organs in our bodies and its capacity of adaptation and regeneration to toxic challenges or surgery has widely been studied [85]. As previously mentioned, we tend to focus on liver injury and cell death forgetting about the remaining functional parenchyma. Most of the time this functional parenchyma could allow us to add value to our diagnosis and prognosis and get a whole picture of what the liver is undergoing facilitating therapeutic decisions.

The elimination of the water-soluble, anionic, FDA-approved dye Indocyanine Green (ICG), through the hepatic parenchyma mainly depends on the blood flow, the hepatocytes integrity and the biliary excretion [86]. Due to its exclusive hepatic clearance, ICG elimination rate has been widely used to assess hepatic blood flow, hepato-splanchnic haemodynamic and liver function [86-88]. Measures of ICG have been clinically used for the assessment of liver function in patients undergoing liver surgery using a spectrophotometry technique by repeat blood sampling which remains the gold standard [86]. They have tried to improve this technique and reduce the cost and time spent as well as the repeat blood sampling by inserting an artery catheter. However, this is still very invasive and not always applicable [86].

The non-invasive and dynamic assessment of ICG clearance *in vivo* has recently been made possible through the development of MSOT. This novel optical imaging modality has been demonstrated to have improved resolution and optical imaging accuracy combined with excellent spatial resolution and deep tissue penetration depth [89,90]. Recent applications have revealed its utility for in vivo imaging in cancer, cardiovascular disease, neurology [91], nephrotoxicity applications [92], as well as in the clinic for the non-invasive imaging of disease activity in Crohn’s disease [93] and the detection of oesophageal varices [94]. MSOT imaging has also been previously used to measure kidney function in adriamycin-induced nephropathy mice model by measuring IRDye 800CW clearance through kidneys in combination with standard biochemical and histological indicators of kidney damage [92]. MSOT measurements of ICG clearance to assess liver function could be used to assess liver impairment in DILI where APAP main histological features are related to hepatocyte death and therefore loss of functional liver mass. The loss of hepatocytes could be assessed by measuring the novel panel of circulating biomarkers accounting for cell death (necrosis and apoptosis) and for inflammatory response combined with ICG kinetics as a marker of liver function. The whole mechanistic picture of liver injury would probably allow for better patient management and therapeutic decisions.

## 8. Hepatic regeneration

### 8.1. Colony stimulating factor 1 (CSF1)

There has been increasing interest in CSF1 as a regeneration marker. Hepatic macrophages are known to mediate innate immune defence mechanisms and promote regeneration (hepatocyte proliferation) following an insult to the liver; and CSF1 is known to illicit control over macrophage numbers [95]. In humans (following partial hepatectomy), elevated circulating CSF1 level can be detected and is thus associated with rapid regrowth/regeneration [95]. A publication from Stutch-field et al. uncovered a correlation between serum CSF1 and patient survival in cases of ALF. The authors suggest that serum CSF1, as a prognostic biomarker, could be a useful tool to stratify patients. Interestingly, the authors also study the effects of CSF1 administration in mice highlighting a translational aspect of the biomarker and moreover identifying CSF1 as potentially holding use in replacement therapy as treatment was able to aid in restoring innate immune function following partial hepatectomy or liver injury [95].

The translatable use, across species, is further emphasized in studies showing successful CSF1-Fc administration to pigs whereby extensive hepatocyte proliferation has witnessed absent liver injury and elevated serum biomarker levels [96].

### 8.2. microRNA-21

Other studies have implicated various miRNAs, such as miR-21, in hepatocyte proliferation [97,98]. Several studies have reported the induction of miR-21 during the first 24 hours of liver regeneration following partial hepatectomy, making miR-21 the miRNA that is most consistently altered during the early stages of regeneration [97,98]. Controversial results were however found in an APAP-induced liver injury model where time-course changes in the expression levels of miRNA-21 were not detectable [99,100]. miR-1A and miR-181 have also been implicated as biomarkers of this kind, as a recent study reports their involvement in the enrichment of selective extracellular RNA that becomes detectable within serum and thus represent biomarkers of active liver regeneration processes in mice [101]. In addition, a previous study reported inconsistent data regarding the role of miRNAs in liver regeneration [18]. Hence, further studies are needed to resolve this issue [99].

## 9. Future outlook

Throughout this article we have intended to evaluate the progress of research into liver injury, particularly how translational circulating and imaging based biomarkers inform on this subject. This review places focus, as does much research, on APAP-induced liver injury as a major occurrence in today’s society. In future applications, it would be interesting to look beyond APAP injury to other forms of drug-induced liver injury and see how each biomarker behaves in these scenarios to determine whether they will hold true as mechanistic tools informing of underlying hepatic injury. It is clear that no one biomarker fits all purposes and that a panel approach must be taken to their development and their qualification must be sought within a defined context of use (Table 1). Moreover, they must provide added value to current tests in the first instance.

**Table 1.** Summary of biomarker characteristics

Biomarkers	Mechanism	Liver specific	Translational	Prognostic	Predicts ALT rise	Early marker	Pre-clinical studies	Clinical studies
miR-122	Cell death	Yes	Yes	No	Yes	Yes	Yes	Yes
HMGB1	Necrosis	No	Yes	Yes	Yes	Yes	Yes	Yes
Acetylated HMGB1	Inflammation	No	Yes	Yes	Yes	No	Yes	Yes
FL K18	Necrosis	No	Yes	Yes	Yes	Yes	Yes	Yes
c K18	Apoptosis	No	Yes	Yes	Yes	Yes	Yes	Yes
GLDH	Mitochondrial	No	Yes	Yes	Yes	Yes	Yes	Yes
mtDNA	Mitochondrial	No	Yes	Unknown	Unknown	Unknown Early stage of research	Yes
Acylcarnitines	Mitochondrial	No	Mouse	Unknown	Unknown	Unknown Early stage of research	Yes
CSF-1	Regeneration Proliferation	No	Yes	Yes	No	No	Yes	Yes
miR-21	Proliferation	No	Yes	No	No	No	Yes	No

In July (FDA) and September (EMEA) 2016 regulators expressed support to the SAFE-T consortium (Safer and Faster Evidence-based Translation; a European network with a focus on many of the safety biomarkers discussed within this review) and the Drug-Induced Liver Injury Network (DILIN) in conjunction with the PSTC (Predictive Safety Testing Consortium; aiming to collaboratively share and validate innovative safety testing methods) highlighting the demand for continued research into DILI biomarkers.

Specifically, regulators detailed encouraging the further development of K18, HMGB1, CSF1 receptor and osteopontin, either alone or in combination for use after an initial diagnosis has been based on ALT levels. It is noted that greater experience is required both clinically and non-clinically to better understand the utility of each of these biomarkers. Furthermore, the statement suggests that miR-122 and GLDH should be studied further, particularly in regards to their performance as liver specific injury biomarkers in patients with acute DILI and compared to non-DILI/healthy controls. Moving forward, there are certainly general and individual biomarker limitations that must be carefully considered and gain future research consideration.

Firstly, and as advised by the regulators, a clinical trial type setup may be required with the enrolment of study subjects from a uniformly early phase through to later stages. In this way, clinical trials hold a unique advantage of data collection and would require the inclusion of a diverse population of both healthy volunteers and patients in order to comprehend biomarker behaviour all throughout injury progression. Translation of clinical trial workflows, such as those discussed for cancer biomarkers, to DILI scenarios has been discussed and this broadly pertains to phase I (assay assessment in healthy and non-healthy scenarios as well as determining healthy reference intervals), phase II (retrospective analysis of biomarkers within clinical samples to direct clinical utility) and phase III confirmatory phase (qualifying the biomarker through large, prospective, multi-center RCT trials) as the traditional drug development paradigm can be called into question by the incorporation of biomarker assessment [102]. Finally, in order to have confidence in adding clinical value through the inclusion of these biomarkers, rapid and reliable measurement must be possible and thus there is an emerging need to develop high-quality point-of-care (POC) diagnostics at a clinical level and moreover with utility in resource-limited settings [103]. The majority of exploratory biomarkers discussed here are currently measured manually in varying research laboratories and with time-consuming and expensive kits, such as mass spectrometry analysis [28], whereas POC assays with high reliability and repeatability, rapid time interval from test to result, that are low in cost allowing high throughput and without mandatory central laboratory testing, are required to fully realise the potential of many promising candidate biomarkers.

## ABBREVIATIONS

AALF: APAP-induced acute liver failure;
Acs: acylcarnitines;
ALD: alcoholic liver disease;
ALF: acute liver failure;
ALT: alanine aminotransferase;
APAP: acetaminophen (or paracetamol);
cK18: caspase-cleaved keratin-18;
CSF1: colony stimulating factor 1;
DAMP: damage associated molecular pattern;
DILI: drug-induced liver injury;
FDA: US Food and Drug Administration;
FL K18: full length keratin-18;
GBCA: gadolinium-based contrast agents;
GLDH: glutamate dehydrogenase;
GSH: glutathione;
HCV: hepatitis C virus;
HCC: hepatocellular carcinoma;
HDE: hepatocyte-derived exosomes;
HMGB1: high mobility group box protein 1;
ICG: indocyanine green;
IDILI: idiosyncratic DILI;
IL: interleukin;
K18: keratin-18;
MRI: magnetic resonance imaging;
miRNA: microRNA;
miR-122: microRNA-122-5p;
MSOT: multispectral optoacoustic tomography;
mtDNA: mitochondrial DNA;
NAPQI: N-acetyl-p-benzoquinonimine;
NAC: N-acetylcysteine;
NAFLD: non-alcoholic fatty liver disease;
NASH: non-alcoholic steatohepatitis;
nDNA: nuclear DNA;
POC: point-of-care;
PSTC: Predictive Safety Testing Consortium;
RAGE: receptor for advanced glycation end products;
ROC-AUC: receiver operator characteristic-area under the curve;
ROS: reactive oxygen species;
SAFE-T: Safer and Faster Evidence-based Translation;
TBL: total bilirubin;
TLR: Toll-like receptors
